# Preliminary Estimates of Mortality and Years of Life Lost Associated with the 2009 A/H1N1 Pandemic in the US and Comparison with Past Influenza Seasons

**DOI:** 10.1371/currents.RRN1153

**Published:** 2010-03-20

**Authors:** Cecile Viboud, Mark Miller, Donald R Olson, Michael Osterholm, Lone Simonsen

**Affiliations:** ^*^Fogarty International Center, National Institutes of Health, Bethesda, MD, USA; ^†^National Institutes of Health; ^‡^Research Director, International Society for Disease Surveillance; ^§^Director of the Center for Infectious Disease Research and Policy (CIDRAP), director of the NIH-supported Center of Excellence for Influenza Research and Surveillance within CIDRAP, a professor in the Division of Environmental Health Sciences, School of Public Health, and an adjunct professor in the Medical School, University of Minnesota and ^¶^Department of Global Health, George Washington University, School of Public Health and Health Services, DC

## Abstract

The on-going debate about the health burden of the 2009 influenza pandemic and discussions about the usefulness of vaccine recommendations has been hampered by an absence of directly comparable measures of mortality impact. Here we set out to generate an "apples-to-apples" metric to compare pandemic and epidemic mortality. We estimated the mortality burden of the pandemic in the US using a methodology similar to that used to generate excess mortality burden for inter-pandemic influenza seasons. We also took into account the particularly young age distribution of deaths in the 2009 H1N1 pandemic, using the metric "Years of Life Lost" instead of numbers of deaths. Estimates are based on the timely pneumonia and influenza mortality surveillance data from 122 US cities, and the age distribution of laboratory-confirmed pandemic deaths, which has a mean of 37 years. We estimated that between 7,500 and 44,100 deaths are attributable to the A/H1N1 pandemic virus in the US during May-December 2009, and that between 334,000 and 1,973,000 years of life were lost. The range of years of life lost estimates includes in its lower part the impact of a typical influenza epidemic dominated by the more virulent A/H3N2 subtype, and the impact of the 1968 pandemic in its upper bound. We conclude that the 2009 A/H1N1 pandemic virus had a substantial health burden in the US over the first few months of circulation in terms of years of life lost, justifying the efforts to protect the population with vaccination programs. Analysis of historic records from three other pandemics over the last century suggests that the emerging pandemic virus will continue to circulate and cause excess mortality in unusually young populations for the next few years. Continuing surveillance for indicators of increased mortality is of key importance, as pandemics do not always cause the majority of associated deaths in the first season of circulation.

## Introduction

The World Health Organization (WHO) has been criticized for responding too forcefully to the 2009 A/H1N1pdm pandemic threat.  One of the charges is that recommendations to develop and distribute vaccine to nations were overzealous, given the perceived “mild” impact of this pandemic. Accusations included that the recommendations to vaccinate had needlessly exposed millions of healthy people to the risk of unknown side-effects of pandemic vaccines. There is concern that the true severity of the 2009 influenza pandemic has been underestimated, and that reporting in the media was inappropriate [1].  

The WHO global estimate of ~16,000 laboratory-confirmed deaths from A/H1N1pdm influenza as of February 26, 2010 [2] appears many fewer than the millions of deaths associated with the three previous pandemics of 1918, 1957 and 1968 [3] [4] [5], or even than deaths associated with seasonal influenza epidemics [6] [7].  However these figures are not comparable for several reasons.  First, the WHO laboratory-confirmed data represent the “tip of the iceberg” of all influenza-related deaths; in contrast, mortality estimates for historic pandemics were based on statistical attribution of excess all-cause mortality during the pandemic period, and are far more inclusive than laboratory-confirmed cases.  In the US alone, the Centers for Disease Control and Prevention (CDC) has used a modeling approach to estimate that 12,000 deaths were associated with the H1N1pdm virus as of February 13, 2010 (range 8,500-17,600)[8].  Second, these ~12,000 deaths are qualitatively different than the ~36,000 seasonal influenza deaths which occur in an average winter in the US [6]-- the majority of pandemic deaths have occurred in children and younger adults [9] [10], while almost all seasonal influenza deaths occur in the elderly [7].    

We propose a framework to generate measurements of mortality impact for the 2009 pandemic comparable to past pandemic as well as to recent epidemic seasons. We first generated an early assessment of the number of deaths in the US, by indexing provisional mortality data from CDC’s 122 Cities surveillance system [11] to final national mortality data from past seasons [7]. We then accounted for the age distribution of laboratory-confirmed 2009 A/H1N1pdm deaths and used  the 122 Cities data to extrapolate the number of Years of Life Lost (YLL) associated with the 2009 pandemic, using the same approach as proposed [4].  We then compare US estimates of YLL for the 2009 pandemic to those for historic pandemics and contemporary influenza seasons.  

## Methods

Age-specific estimates of deaths attributable to the 2009 pandemic are required to calculate the number of years of life lost to the A/H1N1pdm virus.  As vital statistics data are usually not available until 2-3 years after an influenza season or pandemic is over, and only a fraction of cases and deaths are laboratory tested, we used independent sources of data to 1) estimate the age distribution of pandemic-related deaths based on a sample of laboratory-confirmed deaths and 2) apply this age distribution to preliminary estimates of the number of pandemic-related deaths during 2009 in the US, using CDC's timely 122 Cities surveillance data. 

### Estimating age-specific numbers of deaths attributable to influenza.  

The age distribution of deaths was provided by a study published in August 2009 and reporting on 468 laboratory-confirmed A/H1N1 deaths in different countries (Table 1) [9]. The age distribution of deaths was very similar between countries and in agreement with earlier estimates from Mexico [10], with a mean of 37 years. Additional information on the age of 292 laboratory-confirmed A/H1N1 deaths in the US was also available from a CDC publication [12], suggesting a similar mean age of ~40 yrs from April until mid-October 2009. Given that the CDC publication provided only a crude age breakdown, especially among young adults who comprised a substantial proportion of deaths, we present here estimates based on the larger study from August 2009 [9].

    To estimate the overall number of deaths attributable to the pandemic, we relied on the CDC surveillance system for pneumonia and influenza (P&I) deaths -- the 122 Cities provisional mortality surveillance system [13].  The system reports weekly deaths from P&I and all causes based on a large sample of US cities. In the absence of a clear denominator for the population-at-risk in this sample, the CDC has historically used the weekly ratio of P&I deaths (defined as the no. of P&I deaths divided by the total no. deaths) to assess the timing and severity of influenza epidemics with only a few weeks lag time (Figure 1). To convert this proxy indicator into a measure of excess deaths, we compared the ratio from the 122 Cities data to final vital statistics from the National Center for Health Statistics (NCHS), which compiles 100% of US deaths certificates. The comparison relied on 7 past inter-pandemic seasons where both systems overlaped, 1999-2006, as NCHS data lag CDC data by 3-4 years. For comparison purposes, we used both the P&I and all-cause mortality outcomes from the NCHS statistics, as both are traditionally used to measure influenza mortality burden [14] [15].  Although both the 122 Cities and NCHS vital statistics data are available well before 1999, a change in the 122 Cities surveillance system in 1999 resulted in an abrupt rise in the P&I death ratio, which precluded inclusion of earlier years. 

**Figure:**
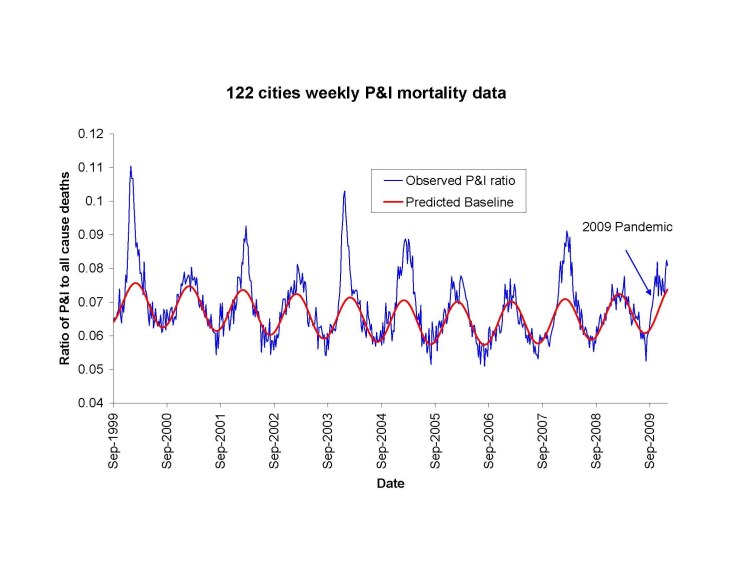
**Figure 1:** Weekly time series of the ratio of deaths from pneumonia and influenza to all deaths, based on the 122 cities surveillance in the US [13] (blue line). The red line represents the expected baseline ratio in the absence of influenza activity.  The area above the baseline during each winter period was calculated and compared with final estimates of P&I and all-cause excess mortality using NCHS final vital statistics.

To derive indicators of influenza mortality burden for each dataset, we fitted traditional Serfling regression models to data in non-epidemic months (excluding Dec-Mar of each year and all months in 2009) to derive baseline deaths in the absence of influenza activity [7] (Fig 1). The influenza attributable burden was calculated as the observed minus baseline during influenza epidemic months and summed over each season. Seasonal excess mortality estimates for P&I and all-cause derived from the NCHS system were then compared against the seasonal excess P&I ratio from the CDC system (Fig 2A & B). A linear regression model was then developed to predict excess P&I deaths as a function of the excess P&I ratio, and a similar approach was used to predict excess all cause deaths. This approach provided estimates of the number of excess P&I and all-cause deaths associated wih the pandemic period May-Dec 2009 in the US, given the excess P&I ratio observed in the 122 cities surveillance during the same period. Using the age distribution from[9], we proportionally generated age-specific estimates of the number of 2009-pandemic deaths. 

**Figure:**
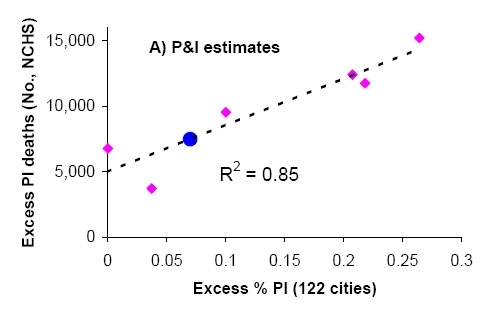

**Figure:**
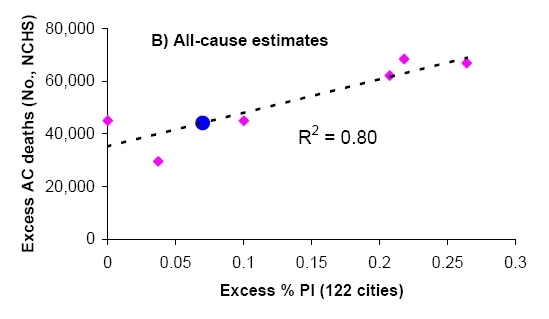
**Figure 2:** Comparison of seasonal influenza mortality burden estimates in the US by two systems: the 122 cities mortality surveillance system [13], and the final mortality data from the national center for vital statistics (NCHS). All estimates are derived from a seasonal regression model applied to weekly mortality data. A) Seasonal excess P&I deaths (NCHS) against seasonal excess ratio of P&I over all cause deaths (122 Cities). B) Seasonal excess all-cause deaths (NCHS) against excess ratio of P&I over all cause deaths (122 Cities). Pink symbols denote historical observations for 1999-2006 and the black line is a linear regression between the 2 mortality indicators. Blue dots illustrate the prediction for the 2009 pandemic, given the model illustrated by the black line and the observed 122 cities’ indicator in 2009.

We note that our excess P&I estimate can be considered as a lower bound of mortality attributable to the 2009 pandemic (as it excludes deaths that are not directly coded as P&I), while our excess all-cause estimate can be considered an upper bound.  The upper bound based on all-cause excess mortality is most similar to the method used historically to generate mortality burden estimates for the 1918, 1957 and 1968 pandemics and inter-pandemic seasons in the US [7] [16] [17].

******

### Estimating Years of Life Lost (YLL) attributable to influenza.  

The concept of using metrics such as Years of Life Lost (YLL) or Disability-Adjusted Life Years lost instead of numbers of deaths is often used to quantify the burden of disease [18] [19].  Following a previously described method [4], we calculated “period expected” YLL by multiplying the number of age-specific deaths attributable to the 2009 pandemic to standard life expectancy at age of death in 2000 from the mid-point of each age category (Table 1). Our algorithm follows a standard approach for estimating Disability Adjusted Life Years [19], but without weighing a social discount rate that favors life saved in the near future, nor using age-specific coefficients to weight deaths in young adults more heavily than in children or seniors.   

****

**Figure:**
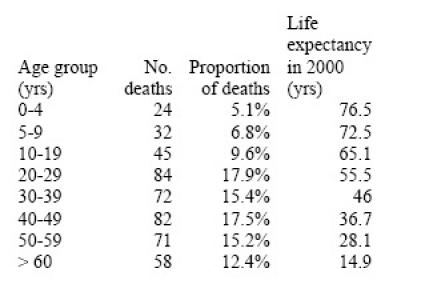
**Table 1:** Age distribution of pandemic-related deaths in 2009 [9] and life expectancy estimates in the US used for calculation of years of life lost** **

## Results

Based on the study reported in August 2009 in [9], more than 85% of laboratory-confirmed A/H1N1 deaths occurred in people under 60 years of age, with a mean age of deaths of 37 years (Table 1). This is in marked contrast to seasonal influenza epidemics where 90% of deaths occur in people over 65 years and the mean age of influenza-related deaths is estimated at 76 yrs.

 The excess mortality estimates based on the final vital statistics compiled by NCHS and the preliminary data provided by the 122 cities surveillance showed strong agreement for the period 1999-2006, despite the low number of seasons available for comparison (R2>0.80 for P&I and all cause excess mortality, Figure 2). Based on the prediction model shown in Figure 2, we estimate that the overall number of deaths attributable to the 2009 pandemic in the US range between ~7,500 (P&I excess deaths) to ~44,100 (all-cause excess deaths) (Table 2). This range includes the latest CDC estimate of 12,000 deaths in the US [8] based on a probability modeling approach developed by Reed et al [20].  It is important to note that Reed et al’s approach differs from the Serfling “excess mortality” approach, and may not take into account influenza-related deaths triggered by exacerbation of underlying chronic health conditions, in the absence of pneumonia [17] [21] [15].

**Figure:**
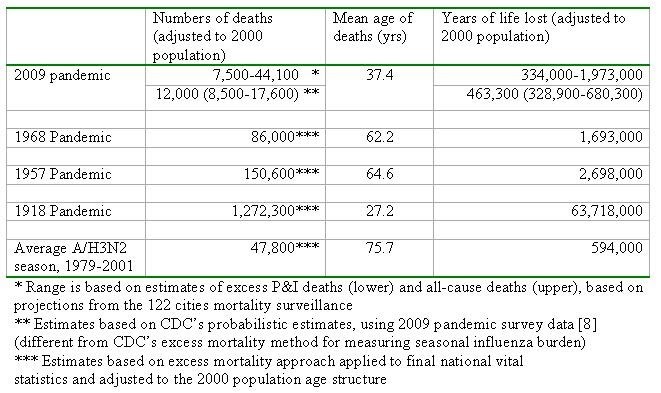
**Table 2:** Estimates of number of deaths, mean age of deaths, and years of life lost attributable to the 2009 pandemic in the US. Estimates for historical pandemics and typical A/H3N2 seasons are provided for comparison purposes.

From this range of mortality estimates, we extrapolate that the pandemic has been responsible for 0.3 to 1.9M of YLL so far in 2009 (Table 2). This range encompasses YLL estimates for a typical influenza A/H3N2 season, as well as for the 1968 pandemic, when estimates are adjusted to the age structure and size of US population in year 2000.  By contrast, the 1957 and 1918 pandemics  were associated with ~30% and ~3000% more YLL than our upper bound for the 2009 pandemic, respectively, when estimates are adjusted to the 2000 population.   Of note however is the particularly young mean age of deaths in the current pandemic, which is substantially lower than in the 1968 and 1957 pandemics, and about 10 year older than in the 1918 pandemic. This unusually young age distribution of H1N1pdm deaths increases the YLL burden of 2009 pandemic greatly compared to the 1957 and 1968 pandemics, despite the relatively lower number of excess deaths estimated so far during 2009 (Table 2).

## Discussion

We have developed a strategy for generating mortality burden estimates comparable across influenza epidemic and pandemic seasons using US mortality surveillance data.  Here we presented an estimated range of Years of Life Lost (YLL) for the 2009 pandemic up to December 31, 2009.  Because of the unusually low mean age of the H1N1 pandemic deaths (37 years), using the YLL metric changes the perception of relatively low impact, both when compared to seasonal influenza (where more than 90% of deaths are in persons aged over 65 years) and to past pandemics.   The resulting range of YLL estimates for the 2009 H1N1 pandemic included in its lower end the burden of an average seasonal epidemic caused by A/H3N2, the most virulent seasonal virus subtype ciruclating in the last 3 decades.  The upper range of YLL estimates for 2009 exceeded the burden of the 1968 pandemic adjusted to the year 2000 population, a pandemic with a relatively high mean age of death at 62 years. We believe our YLL approach more accurately reflects the qualitative differences between pandemic and inter-pandemic influenza mortality, using methods frequently used in health outcome evaluations.

We derived the lower end of the YLL range using the conservative assumption of counting only deaths coded as pneumonia and influenza. This was most similar to the approach used to quantify the burden of the 1918 pandemic, the only other pandemic we are aware of that had a low median mortality age (27 years). Interestingly in 1918, 95-100% of the influenza burden was captured by deaths coded as pneumonia, influenza, and bronchitis, based on data from NYC and Copenhagen[22] [23]. By contrast, the higher end of our YLL range estimate for the 2009 pandemic derives from methods used to estimate burden of the 1957 and 1968 pandemics and seasonal epidemics. This method accounts for influenza-related deaths in part attributable to underlying high risk conditions, but occurring in excess of a normal baseline mortality level during the influenza activity period.  Given the unseasonal nature of this pandemic, it is difficult to determine what best describes the 2009 pandemic mortality burden at this time – but final estimates will resolve this once national vital statistics data are available.

****

We believe that the use of qualitative designations such as mild, moderate and severe in describing the health impact of seasonal and pandemic influenza is insufficient, and possibly inappropriate. This terminology describes a pandemic from a single outcome measure; namely an estimate of all direct and contributing deaths. However, the substantial difference in the age distribution of pandemic-related deaths, the number of patients requiring intensive care, and the loss to society in work productivity when a younger age population is disproportionately impacted, supports the use of alternative measures to describe the burden of influenza pandemics and compare with typical influenza seasons. While we have not identified a single outcome measure that can simultaneously account for all the above variables, we believe that using the YLL metrics is the best single measure at this time. From that perspective,  the 2009 pandemic had a significant impact on the world’s health from April through December 2009..

Based on US mortality surveillance data, we conclude that the YLL burden of the 2009 pandemic may in fact be as high as for the 1968 pandemic – but that at this time the assessment is still tentative.  More H1N1 pandemic waves are likely to occur over the next seasons, increasing the cumulative burden of the emerging virus, and severity may also change over time.  For example, the 1918 1^st^ wave was relatively mild compared to the devastating autumn 1918 wave that killed 2% of the global population [22] [23] [24].  In Europe and Asia, the majority of deaths associated with the 1968 pandemic occurred a full year after the pandemic virus first began circulating [15].   Only several years after the emergence of the H1N1-pandemic, when national vital statistics become available, will we know the full extent of the severity of this pandemic in the US. Finally, the mortality burden of the 2009 pandemic remains poorly studied in other countries and potential geographical differences in excess mortality rates remain unclear.  An early assessment using Mexican data suggested the severity may have been similar to the 1957 pandemic, a pandemic believed to have caused 2 million deaths worldwide.  However, data from Europe and the US, including the present study, suggest a milder impact there similar to what occurred in the first year of the 1968 pandemic.  Geographical variability in mortality impact was also seen during the 1918 pandemic, where for example Scandinavian cities and NYC experienced a mild 1^st^ wave, followed by the catastrophic second wave in the autumn [23] while cities like Geneva [25] and Madrid [26]experienced their severe impact in the first wave.   

In conclusion, based on US data for May-December 2009, we estimate that the H1N1 pandemic was associated with an impact ranging from that of a virulent influenza A/H3N2 season to that of the 1968 pandemic, when one takes into account the markedly young age distribution of influenza-related deaths in occurring in 2009. The 2009 H1N1 virus may replace one or more A type influenza viruses and cause additional waves either late this season or the following winter, potentially with additional mortality impact to come.  Given the historic record of changing disease severity geographically and over time, it is critical to keep up vigilance with this emerging pandemic virus, and continue vaccination efforts and other preparations to protect the population.  The recent recommendation to include the pandemic H1N1 virus antigen in the seasonal vaccine formulation for the next winter is a good step towards this goal. 

## Funding Information

This work was supported by the intra-mural research program of the Fogarty International Center, National Institutes of Health (CV, MM); the RAPIDD program of the Science & Technology Directorate, Department of Homeland Security and Fogarty International Center, NIH (LS); and the National Institute of Allergy and Infectious Diseases, NIH, under contract HHSN266200700007 (MO).

## Competing Interests

The authors have declared that no competing interests exist.
